# Neuronavigation-guided Judet screw technique for C2 pedicle fractures: how I do it

**DOI:** 10.1007/s00701-025-06476-w

**Published:** 2025-03-12

**Authors:** Giuseppe Maria Vincenzo Barbagallo, Francesco Certo, Carmelo Vitaliti, Giulio Bonomo

**Affiliations:** 1 Department of Neurological Surgery, Policlinico “G. Rodolico-S. Marco” University Hospital, Viale Carlo Azeglio Ciampi, 95121 Catania, Italy; 2 Department “G.F. Ingrassia”, Section of Neurosciences, University of Catania, Catania, Italy

**Keywords:** Neuronavigation, Judet technique, C2 pedicle fracture, C2 pedicle screw fixation, Minimally invasive surgery, Atlanto-axial instability

## Abstract

**Background:**

Atypical Hangman’s fractures may involve bilateral C2 pedicle fractures. Surgical fixation is often required to prevent instability and neurological impairment. The Judet technique, involving transpedicular screw fixation, offers a targeted approach to stabilize C2 pedicle fractures while preserving cervical motion.

**Method:**

This article presents a neuronavigation-guided modification of the Judet technique for C2 pedicle screw placement. Advanced intraoperative computed tomography (CT) imaging, virtual trajectory planning and intraoperative navigation guidance provide surgical precision and patient safety.

**Conclusion:**

Neuronavigation can improve the classical Judet technique, enhancing clinical safety and accuracy in fixation of C2 pedicle fractures. This minimally invasive/mini-open approach preserves C1-C2 mobility and reduces complications.

**Supplementary information:**

The online version contains supplementary material available at 10.1007/s00701-025-06476-w.

## Introduction

Traumatic spondylolisthesis of the axis, commonly referred to as a Hangman’s fracture, represents one of the most frequent fractures of the C2 vertebra, second only to fractures of the odontoid process. [3] This injury involves a bilateral fracture of the pars interarticularis of C2, leading to instability between the vertebral body and the posterior elements of the axis. [3] Bilateral pedicle fractures of C2 are classified as "atypical" fractures, with only a limited number of cases reported in the literature. [8] For unstable fractures, particularly in cases involving significant vertebral displacement or risk of spinal cord injury, surgical spine fixation is often necessary. [7] Among surgical options, posterior transpedicular screw fixation offers direct stabilization of the C2 vertebra. Originally described by Leconte and later refined by Judet, the “Judet technique” involves placement of cancellous lag screws through the C2 pedicles. [5, 6, 9] This technique is highly effective in selected cases, with minimal soft tissue damage, offering a minimally invasive approach, which preserves motion and minimizes complications. [1, 2].

**Fig. 1:**
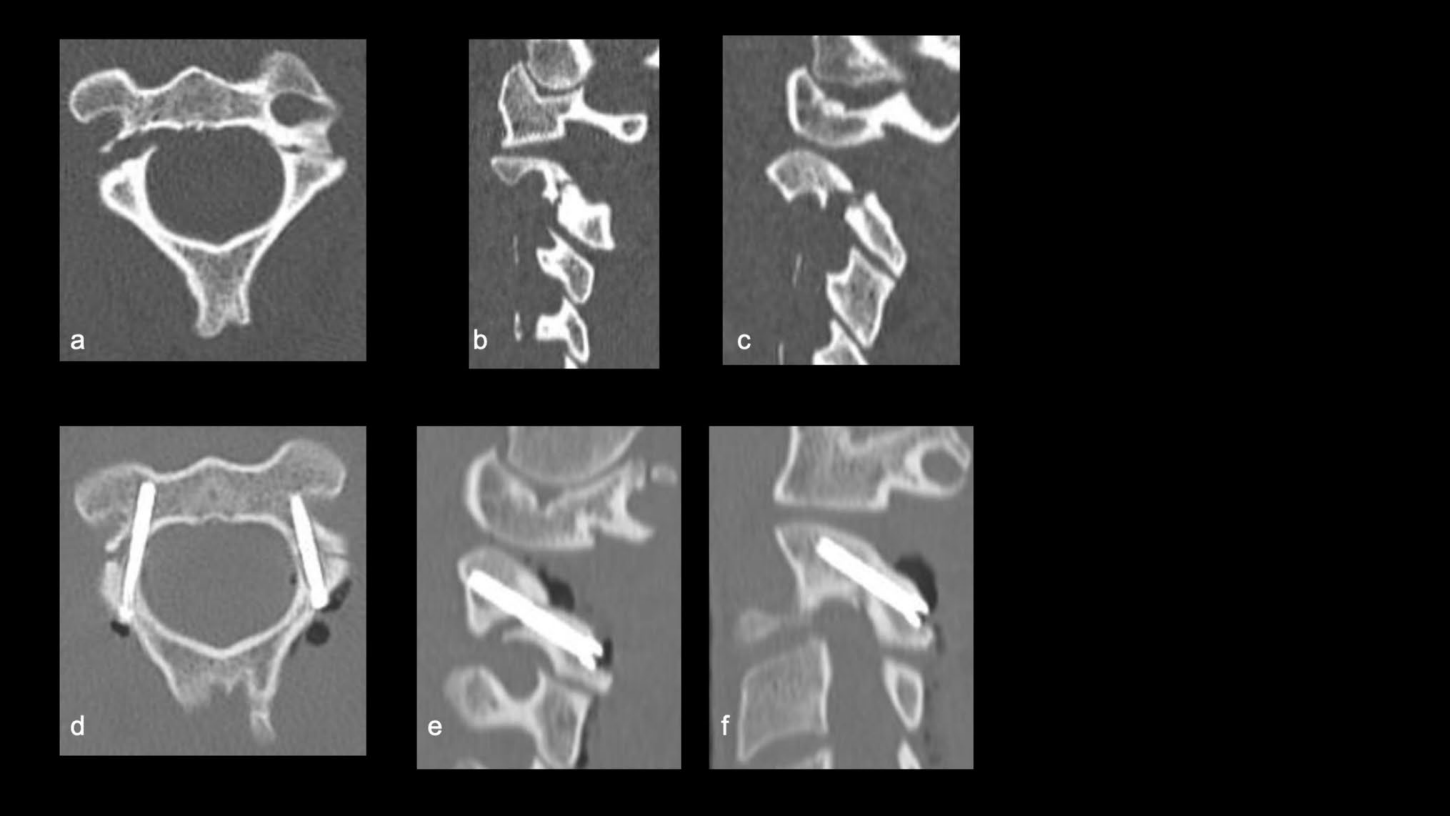
Preoperative axial (**a**) and sagittal (**b**, **c**) cervical spine CT scan showing a fracture of the two pedicles of C2. Postoperative axial (**d**) and sagittal (**e**, **f**) cervical spine CT images demonstrating C2 transpedicular screws (Judet technique) with reduction of the fracture gap

**Fig. 2:**
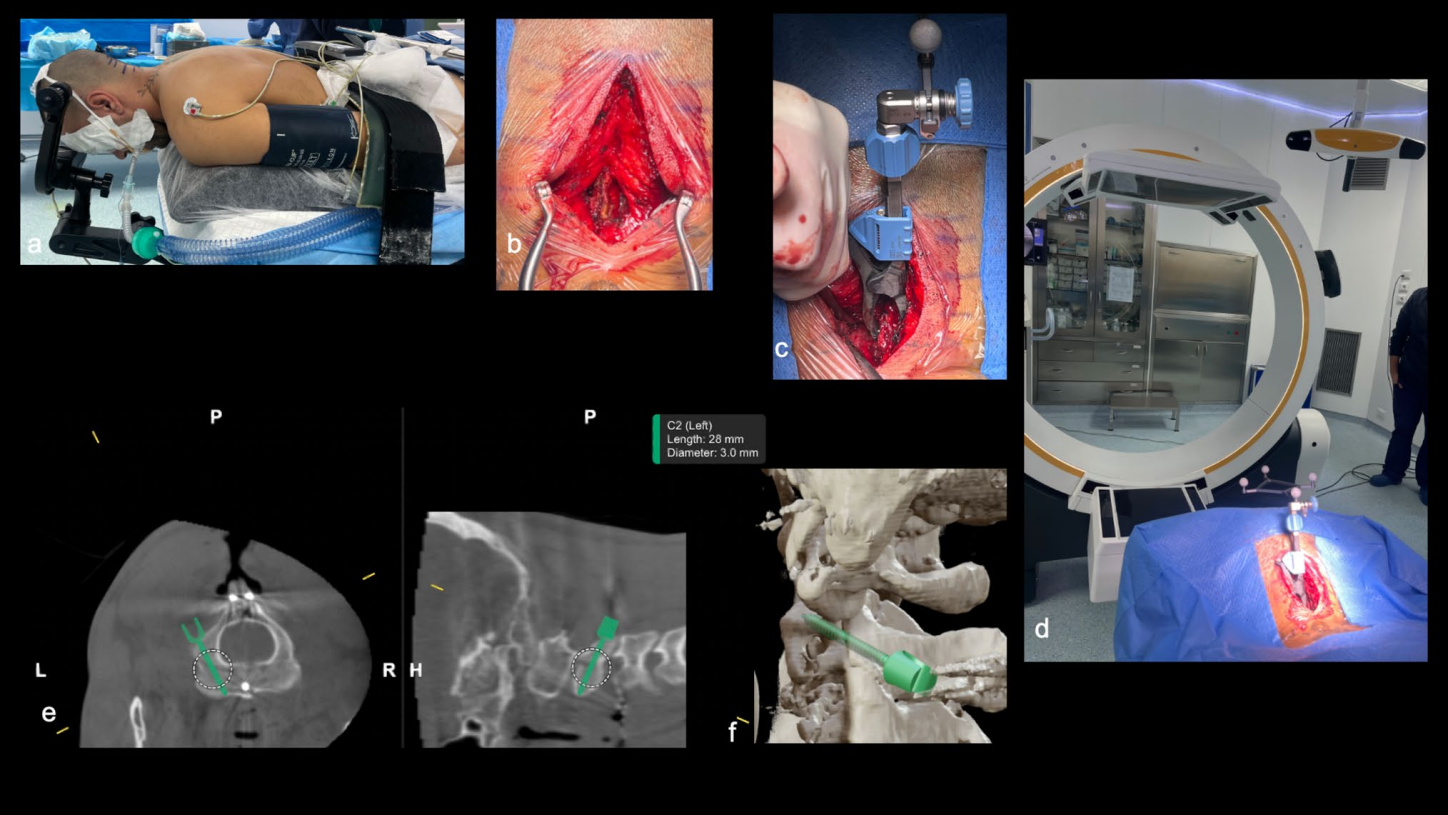
**a** Patient positioning in the operating room in a prone position using a three-point radiolucent head holder. **b** Midline posterior cervical skin incision, exposure of the C2 spinous process, laminae, and articular processes, followed by fixation of a navigation reference to the C2 spinous process (**c**). **d** Operating room setup with the LoopX imaging system and navigation camera (Brainlab) aligned to the reference secured to the patient. **e** Preoperative virtual planning on the Brainlab neuronavigation workstation, using the LoopX CT scan to define the trajectory, entry point, and dimensions of the screws for the unilateral fracture of the left pedicle (with CT images maintaining natural left–right orientation); 3D reconstruction view (**f**)

**Fig. 3:**
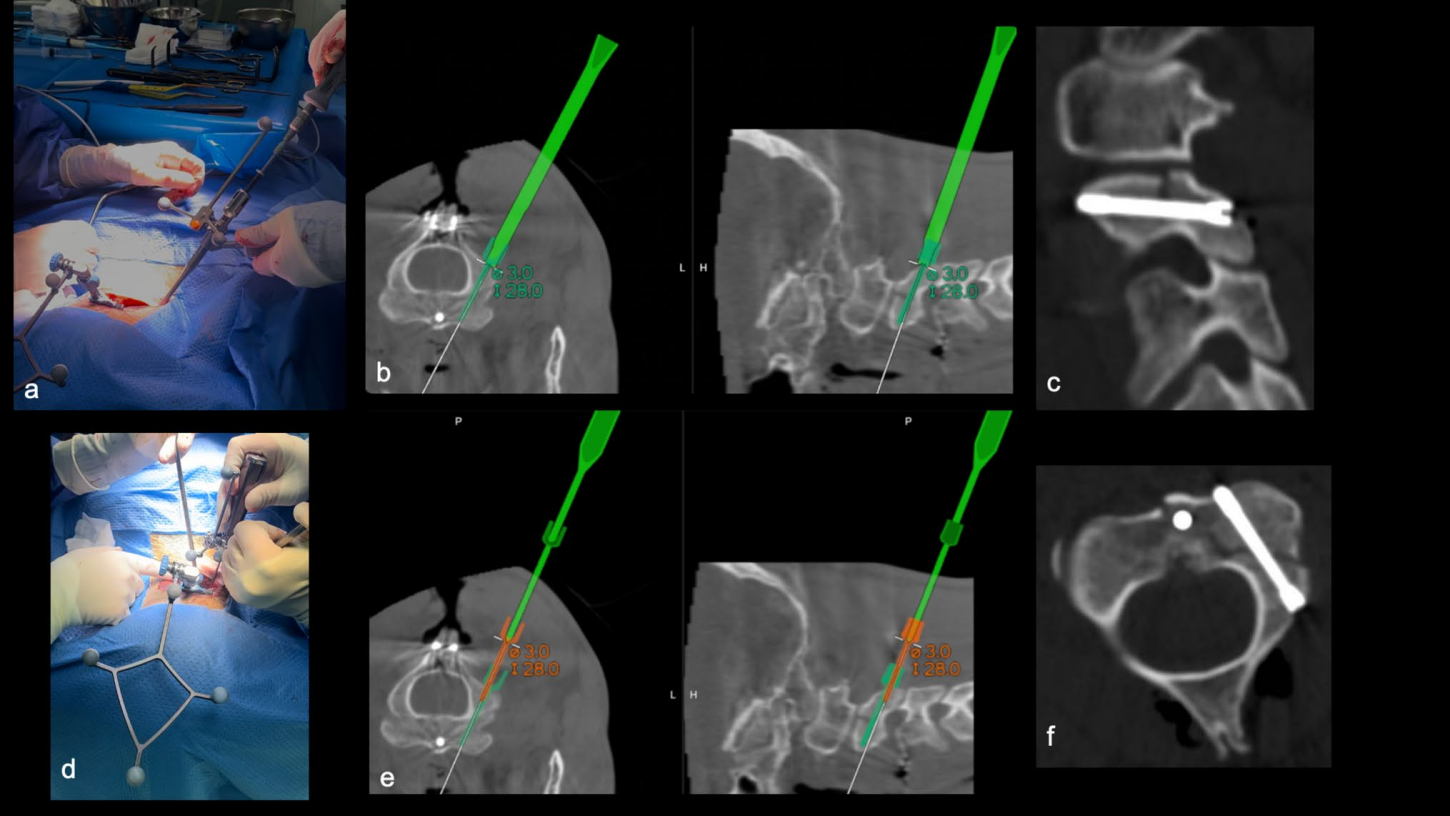
**a** Intraosseous drilling is performed through a neuronavigated working channel, guided in real-time by the neuronavigation system; **b** the trajectory follows the pre-planned path. **d** A navigated screw holder is used to place the screw, again guided by the pre-planned imaging (**e**). Intraoperative cervical spine CT images confirm the correct positioning of the screw in the left pedicle of C2, shown in sagittal (**c**) and axial (**f**) views

We present our "How I do it" approach, utilizing neuronavigation to enhance the precision of C2 pedicle screw placement and reduce the risk of neurological and vascular complications. This advanced technology allows for more accurate identification of anatomical landmarks, ensuring safer and effective fixation in bilateral (Fig. 1) and monolateral (Figs. 2 and 3) C2 pedicle fractures, as Hangman’s fractures and also in complex C2 fractures (combination with odontoid process fracture).

## Relevant surgical anatomy

In C2 pedicle screw fixation, exposing the screw entry points typically follows the identification of the C2 lamina and articular process. The entry point is located 2 mm lateral to the pars midpoint along the cranial edge of C2 lamina. After identifying this point, surgeons try to guarantee trajectory accuracy by visualizing the medial and superior borders of the pedicle. [4]

One of the most critical anatomical considerations is the relationship between the vertebral artery (VA) and the C2 pedicle. The VA passes through the transverse foramen and along the vertebral artery groove, lateral to the pedicle. [9] Anatomical variations, particularly high-riding vertebral arteries, significantly increase the risk of injury during screw placement, with reports of injury rates around 8.2%. [2] Thorough knowledge of these variations is crucial for avoiding complications. Preoperative imaging evaluation provides safer screw placement, especially in complex cases. Neuronavigation is highly valuable in this context as it enhances the surgeon’s ability to precisely identify both the anatomical landmarks and the entry point and to guide screw trajectory. [4]

## Description of the technique

The patient is positioned prone, with a radiolucent 3-pin head-holder, in the "military tuck" position (Fig. 2a). Anti-Trendelenburg positioning is mandatory to facilitate surgical exposure and reduce venous bleeding. Initially, 2D fluoroscopy is performed to verify the alignment and positioning of the bony fragments (e.g., also in cases of concurrent odontoid process fracture). The surgical field is prepped and draped. A small, midline, skin incision is made at the level of C2 spinous process (mini-invasive/mini-open). The cervical fascia is incised and the spinous process, laminae, and articular processes of C2 are skeletonized using monopolar (Fig. 2b) Once bony exposure is complete, the navigation reference frame is securely attached to the C2 spinous process (Fig. 2c). The LoopX system (Brainlab, Munich, Germany) or the BodyTom Computed Tomography (CT) scanner (Neurologica, Samsung, US) are used to obtain an initial CT scan of the cervical spine, enabling automatic registration with the Brainlab navigation systems (Fig. 2d). Validation of all trackable instruments is mandatory before surgery begins. Navigation accuracy is verified using clear anatomical landmarks. The virtual screw trajectory is planned on the navigation station using the CT scan acquired earlier, with virtual 3D screws of the desired dimensions (length and diameter) (Fig. 2e, f). Trajectory is confirmed using the navigation pointer, as well as adequacy of bony exposure. A navigated tubular guide is placed and held manually by a surgical assistant. Through this working channel, the drill is used to prepare the screw trajectory inside the fractured C2 pedicle and K-wires are then inserted. (Fig. 3a, b). Drilling must pass through the pars and into the fractured pedicle stumps. A navigated screw driver is used to insert the screw over the guidewire, with obvious tactile feedback indicating the crossing of the fracture line (Fig. 3d, e). Finally, a 3D-reformatted CT scan with the LoopX or BodyTom system is performed to confirm correct positioning of the screws and reduction of the fracture line (Fig. 3c, f). The navigation reference is removed and the cervical fascia and skin incisions are closed with running sutures and metal clips, respectively.

## Indications

Minimally invasive/mini-open neuronavigation-guided posterior C2 transpedicular “Judet screw fixation” is a safe and effective technique used to manage C2 instability. This approach reduces bleeding and muscular damage while providing lower risks of vertebral artery injury and increased accuracy compared to traditional C1-C2 fixation. Additionally, by avoiding C1-C2 fixation it also preserves rotational head motion, offering a significant functional advantage. Yet, by minimizing the extent of cervical spine exposure, the proposed technique also helps to decrease postoperative pain and to accelerate patient recovery.

## Limitations

The main limitation of this technique is related to the need for spinal navigation technology, which, although increasingly available in neurosurgical operating rooms, is not yet universally spread or accessible. Spinal neuronavigation is also useful to assist less experienced surgeons in accurately placing screws by enabling meticulous preoperative planning and guiding them through anatomical trajectories. Compared to percutaneous, navigated, tubular approaches with the reference frame attached to the head holder, this technique requires a slightly longer skin incision (mini-open) with moderately greater muscle exposure. However, it offers the advantage of direct anatomical control of both entry points and screw trajectory through the bony stumps of the fractured C2 pedicle, providing a higher degree of surgical precision.

## How to avoid complications

Reliability of neuronavigation should be continuously verified throughout the surgical procedure, step by step, in order to confirm technical reliability and real correspondence between intraoperative CT-guided navigation images and exposed bony anatomical structures. 3D-reformatted CT scan allows accurate monitoring of screw placement. A thorough knowledge of vascular anatomy, as well as of traditional surgical techniques, entry points and trajectories for C2 pedicle screw fixation remains essential.

## Specific information for the patient

Patients should be counseled about potential risks, including vertebral artery injury with neurological deterioration, infection, hematoma or misplaced screw insertion. Additionally, they should be informed about the possibility of pseudoarthrosis.

## Summary of key points

1. C2 pedicle fractures can occur as atypical Hangman’s fractures or as part of complex fractures, also involving the odontoid process.
2. The Judet technique involves placing cancellous lag screws through the C2 pedicles, offering direct C2 pedicle reconstruction and fixation, avoiding C1-C2 fixation with consequent loss of neck motion.
3. Minimally invasive/mini open neuronavigation-guided posterior C2 pedicle screw fixation is a safe and effective technique for managing atlanto-axial instability.
4. Anatomical variations of the vertebral artery must be ruled out preoperatively (CT-angiography) to avoid vascular complications.
5. The Judet technique preserves rotational head movement, a key functional advantage over C1-C2 fixation.
6. It enables faster recovery and shorter hospital stays, as it minimizes postoperative discomfort and rehabilitation time compared to C1-C2 fixation.
7. Expertise in traditional C2 pedicle screw placement remains essential.
8. Verification of navigation accuracy, using known bony landmarks and intraoperative 3D-reformatted CT imaging, is crucial to provide safety and screw placement accuracy.
9. Patients should be informed about surgical risks, including artery injury, infection, and pseudoarthrosis.
10. The main limitation seems to be related to availability of spinal navigation technology, which is not yet universally accessible.

## Supplementary information

Below is the link to the electronic supplementary material.

ESM 1 (MP4 172 MB)

## Data Availability

No datasets were generated or analysed during the current study.

## Abbreviations

CT: Computed tomography
VA: Vertebral artery
